# The ‘myth of Hydroxychloroquine (HCQ) as post-exposure prophylaxis (PEP) for the prevention of COVID-19’ is far from reality

**DOI:** 10.1038/s41598-022-26053-w

**Published:** 2023-01-07

**Authors:** Deba Prasad Dhibar, Navneet Arora, Deepak Chaudhary, Ajay Prakash, Bikash Medhi, Neeraj Singla, Ritin Mohindra, Vikas Suri, Ashish Bhalla, Navneet Sharma, Mini P. Singh, P. V. M. Lakshmi, Kapil Goyal, Arnab Ghosh

**Affiliations:** 1 Department of Internal Medicine, PGIMER, Chandigarh, India; 2 Department of Pharmacology, PGIMER, Chandigarh, India; 3 Department of Virology, PGIMER, Chandigarh, India; 4 Community Medicine and School of Public Health, PGIMER, Chandigarh, India; 5 Department of Internal Medicine, F-Block, Neheru Hospital PGIMER, Chandigarh, 160012 India

**Keywords:** Randomized controlled trials, Epidemiology, Viral infection

## Abstract

The efficacy of Hydroxychloroquine (HCQ) as post-exposure prophylaxis (PEP) for the prevention of COVID-19 was contentious. In this randomized control double-blind clinical trial, asymptomatic individuals with direct contact with laboratory-confirmed COVID-19 cases were randomized into PEP/HCQ (N = 574) and control/placebo (N = 594) group. The PEP/HCQ group received tablet HCQ 400 mg q 12 hourly on day one followed by 400 mg once weekly for 3 weeks, and the control/Placebo group received matching Placebo. The incidence of COVID-19 was similar (p = 0.761) in PEP [N = 24 out of 574, (4.2%)] and control [N = 27 out of 594, (4.5%)] groups. Total absolute risk reduction for the incidence of new-onset COVID-19 was -0.3% points with an overall relative risk of 0.91 (95% confidence interval, 0.52 to 1.60) and the number needed to treat (NNT) was 333 to prevent the incident of one case of COVID-19. The study found that, PEP with HCQ was not advantageous for the prevention of COVID-19 in asymptomatic individuals with high risk for SARS-CoV-2 infection. Though HCQ is a safer drug, the practice of irrational and indiscriminate use of HCQ for COVID-19 should be restrained with better pharmacovigilance.

## Introduction

The novel corona virus (SARS-CoV-2) pandemic affected more than 575 million people worldwide with more than 6.3 million loss of life, as of 2nd August 2022^1^. Globally, after the USA India is the second worst devastated country from COVID-19, with more than 44 million affected population and more than 526 thousand fatalities, as of 2nd August 2022^2^. The clinical presentation of the COVID-19 ranges from asymptomatic or mild influenza-like illness, isolated or associated thrombotic/ischemic events to severe pneumonia with acute respiratory distress syndrome (ARDS) and subsequent secondary infection, sepsis, and multi-organ dysfunction syndrome (MODS) contributing to the mortality^3^.

When the study was carried out, there were no definitive therapeutic drugs available for the treatment of COVID-19. COVID-19 patients used to be managed with symptomatic and supportive care only, which includes mechanical ventilation, antibiotics for secondary infection, anti-inflammatory or immune-modulators for the hyperimmune response, and blood thinner to combat thrombotic events^4^.

Many drugs, Ribavirin, Lopinavir, Remdesivir, Molnupiravir, Chloroquine**,** Hydroxychloroquine, Azithromycin, Ivermectin, Tocilizumab, were explored or investigated but the majority of them turned out to be ineffective or partially effective for the treatment of COVID-19 and prevention of mortality^5–7^. In late 2021, oral antiviral drugs like, Paxlovid, Molnupiravir received emergency use authorization from the US Food and Drug Administration (FDA) for mild to moderate cases of early COVID-19, as in randomized control clinical trials (RCT) it was found to reduce the risk of hospitalization or death^8,9^. Sotrovimab, a monoclonal antibody also received emergency use authorization from the FDA for mild to moderate COVID-19 cases but was later found to be ineffective against the predominant Omicron BA.2 variant^10,11^. But the effectiveness of these drugs as PEP for the prevention of COVID-19 is yet to be proven^8–11^. Isolation and quarantine measures are implemented for suspected or proven SARS-CoV-2-infected individuals to confine the spread of COVID-19. Mass vaccinations, post-exposure prophylaxis (PEP), face mask, social distancing, personal or hand hygiene are being enforced for the prevention of COVID-19.

Hydroxychloroquine sulfate (HCQ), a chloroquine analog, was explored both for treatment as well as prevention of COVID-19 because of its potential anti-viral properties^12,13^. Though the evidence indicated that HCQ had very little or no therapeutic advantage in hospitalized COVID-19 patients^14,15^, the role of HCQ as post-exposure prophylaxis (PEP) for the prevention of COVID-19 was contentious or conflicting in our national settings.

Initially, in March 2020 and subsequent revision with supporting evidence in May 2020, the Indian Council of Medical Research (ICMR) advisory endorsed HCQ prophylaxis for the individuals at risk for SARS-CoV-2 infection, which included all asymptomatic health care workers (HCW) involved in suspected or confirmed COVID-19 patient-care and all asymptomatic household contacts of laboratory-confirmed COVID-19 cases^16,17^. This encouraged us to carry out an open-level, non-randomized study on HCQ as PEP for the prevention of COVID-19 in asymptomatic high-risk household contact of the laboratory-confirmed COVID-19 cases during March to July 2020. The outcome was very promising, showing an absolute risk reduction of around 9% for prevention of COVID-19 in participants who received PEP with HCQ as compared to the control group which did not receive HCQ and there were no serious adverse events^18^. HCQ is comparatively a safer drug, used for lifelong therapy in rheumatology patients with few side effects, which allows for a higher dose without significant side effects or drug interactions.

But there were contradictory scientific evidence to prove or disprove the efficacy of HCQ as post-exposure chemo-prophylaxis for the prevention of COVID-19 in individuals at risk for SARS-CoV-2 infection^19,20^. As per Boulware et al., HCQ was not advantageous as post-exposure prophylaxis (PEP) for the prevention of COVID-19^21^. But in this study majority of the participants were HCW. Simultaneously, other published studies had come in support of HCQ prophylaxis as the potential preventive or therapeutic measure against COVID-19^13,22–25^, all though these studies were predominantly observational in nature. At the same time, there was no national-level/domestic randomized control clinical trial (RCT) available to verify the efficacy of HCQ as PEP for the prevention COVID-19 in Indian population.

Household individuals in direct contact with COVID-19 patients are at extreme risk for SARS-CoV-2 infection. HCW represented a large part of the sample in the majority of the concluded studies and there were still limited clinical trials regarding the HCQ prophylaxis for the prevention of COVID-19 in non-HCW individuals who were at risk of SARS-CoV-2 infection. Through this RCT we made an effort to evaluate the efficacy of HCQ as PEP for the prevention of COVID-19 in asymptomatic household individuals in direct contact with laboratory-confirmed COVID-19 cases, who were not HCW. As a research institute of national interest, it brought us enormous scope to generate such strong evidence which could be utilized in reforming nationwide and global guidelines for the battle against COVID-19 pandemic.

## Methods

The methodology followed in this study was similar to our previously published institutional study, regarding HCQ prophylaxis for the prevention of COVID-19^18^.

### Aims of the study

To evaluate the efficacy of Hydroxychloroquine (HCQ) as post exposure prophylaxis (PEP) for the prevention of COVID-19 in asymptomatic high-risk household individuals in direct contact with the laboratory-confirmed COVID-19 cases.

### Site of study

It was a single center study. The individuals with history of high-risk direct contacts with COVID-19 case were screened and enrolled for the study in the COVID-19 screening clinic at Emergency medical outpatient department (EMOPD) and Communicable disease ward of the Post Graduate Institute of Medical Education and Research (PGIMER), Chandigarh, India. The study was executed under cooperation between the Department of Internal Medicine, Virology, Pharmacology, and Community Medicine & School of Public Health of the institution.

### Study design

In this randomized control double blind clinical trial, asymptomatic individuals with direct contact with laboratory-confirmed COVID-19 cases were screened for enrolment according to the inclusion and exclusion criteria and randomized into PEP/HCQ and control/placebo group after getting written informed consent. The participants were family members, relatives, friends or colleagues who were living with or spent hours/days with COVID-19 patients without following any personal protective measures^18^. They visited EMOPD, accompanying their patient with a medical emergency that later on turned out to be COVID-19 related. The duration of unprotected exposure to COVID-19 patients varied from few hours to few days. The PEP/HCQ group received tablet HCQ and control group received matching placebo instead of HCQ. Both the groups were advised for home quarantine for 2 weeks along with social distancing and personal hygiene, and followed up for 4 weeks, physically or telephonically, as and when indicated^18^. The trial was registered with clinicalTrial.gov (clinicaltrials.gov PRS ID: NCT04858633, 26/03/2021).

### Study duration

The clinical trial was executed from the month of March to July 2021 during the second wave of the COVID-19 pandemic. The enrollment period was from 22nd march 2021 to 17th June 2021. The Delta variant of COVID-19 was an emerging predominant variant during that time.

### Inclusion and exclusion criteria

Regardless of gender with age ≥ 18 years, all asymptomatic individuals with direct contact with laboratory-confirmed COVID-19 cases were included in the study^18^. Persons refusing HCQ prophylaxis, with a history of hypersensitivity to HCQ or 4-aminoquinolone derivatives, and individuals with already diagnosed retinopathy, cardiac arrhythmia, G6PD deficiency, psoriasis and pregnancy were excluded from the study^18^. Also, persons with COVID-19-related symptoms, HCW related to suspected or confirmed COVI-19 and individuals with baseline abnormal ECG or who had already received the COVID-19 vaccine were disallowed from participating in the trial. Any person working in any hospital or patient care setting was considered as HCW and excluded from the study.

### Method and intervention

In this randomized control double blind clinical trial, the participants were randomly assigned into either PEP/HCQ group or control/placebo group, after getting written informed consent. The PEP/HCQ group received tablet HCQ 400 mg q 12 hourly on day one, followed by 400 mg once weekly for 3 weeks (total 5 tablets and collective dose of 2000 mg). The control/Placebo group received matching Placebo, 1 tablet q 12 hourly on day one, followed by one tablet once weekly for 3 weeks (total 5 tablets) instead of HCQ. Both groups were advised with standard care in the form of home quarantine for 2 weeks along with social distancing, and personal hygiene. The dose of HCQ for the study intervention was reached according to the ICMR recommendation for PEP with HCQ^13,17^. The promising antiviral and anti-inflammatory characteristics of HCQ, being inexpensive, better oral-bioavailability, higher concentrations in the lung relative to plasma, and fair safety profile backed the preparation of this nationwide advisory^13,17^. The drug was distributed in person to the participants during visiting COVID-19 screening clinic and the participants received the 1st dose of the drug under direct observation therapy (DOT) whenever feasible. The time interval from recruitment/confirmed exposure to intervention/first dose of HCQ was < 24 h. The participants were followed up for 4 weeks, physically or telephonically as and when indicated. They were questioned regarding the appearance of any COVID-19-related symptoms like fever, cough, sore throat, shortness of breath, diarrhea, myalgia, head ache, anosmia, or any adverse drug event. During the course, nasopharyngeal and or throat swab were taken from the participants and processed for reverse transcription polymerase chain reaction (RTPCR) to identify SARS-CoV-2 RNA for establishing the diagnosis of COVID-19^18^. Swab for RTPCR were taken when any participants became symptomatic and by the 5–14 days of contact in case of asymptomatic participants through in-hospital visit or at regional ICMR-approved COVID-19 laboratory.

The COVID-19 cases were defined and categorized as follows^18^. Participants with RTPCR positive for SARS-CoV-2 RNA and with or without COVID-19-related symptoms were defined as definite COVID-19 cases. The participants with new-onset COVID-19-related symptoms, but RTPCR negative for SARS-CoV-2 RNA or couldn’t be performed for any reason were defined as a probable COVID-19 cases. Definite and probable COVID-19 cases collectively were defined as COVID-19 cases. Asymptomatic participants with RTPCR negative for SARS-CoV-2 RNA or couldn’t be performed for any reason were defined as non-COVID-19 cases. Incidence of COVID-19, definite COVID-19 and probable COVID-19 in previously asymptomatic participants were compared between the HCQ and placebo groups^18^. Standard analysis of blood or chest X-ray at upfront was not performed as the participants were asymptomatic and clinically healthy and as potential source of SARS-CoV-2 there was additional risk for spreading the virus to the healthy individual. ECG was offered to the participants as per institutional ethics committee recommendation to exclude participants with abnormal baseline ECG like QT prolongation, arrhythmia, ST segment or T-wave changes.

The participants, who were diagnosed to be COVID-19 cases, were instructed for continuing home quarantine along with symptomatic medications. They were followed up telephonically for any disease progression and offered admission at the COVID-19 center of the institution, PGIMER, Chandigarh, for further treatment according to the institutional COVID-19 management protocol.

### Outcome

Primary outcome was the incidence of COVID-19 cases, definite COVID-19 cases & probable COVID-19 cases. Secondary outcomes were 1.new onset symptoms of COVID-19, 2. Compliance to the advised therapy, 3. Incidence of adverse drug reaction (ADR).

### Sample size and statistical analysis

**Figure 1:**
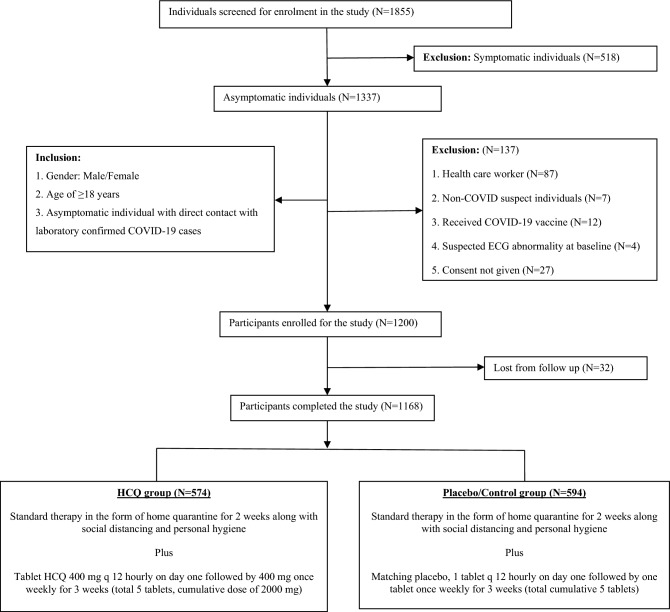
Study design, screening and enrollment.

As per our institutional pilot study during the first wave of the COVID-19, the incidence of COVID-19 was considerably (P = 0.033) lower in the PEP group (10.6%) that received HCQ prophylaxis compared to the control group (19.5%) with absolute risk reduction of –8.9% points^18^. However one interesting aspect was observed through the second wave of COVID-19 was, the positivity rate among the high risk contact of COVID-19 patients reduced significantly due to increased awareness about the disease among the common people and better adherence to the use of face mask, maintaining adequate social distancing and hand hygiene at home itself. Many patients’ attendants had received the COVID-19 vaccine also, interrupting the chain of transmission further. After deliberation with the data safety monitoring board (DSMB), anticipating about 50–60% reduction in the positivity rate, at least 8–10% incidence of COVID-19 in control group was expected as compared to 4–5% incidence rate in PEP group. The calculated sample size was around 900–1100, with an 80% (power) chance of detecting a significant difference between the two groups at a one-sided 0.05 significance level (α-error of 5%). Considering around 10% dropout, the total sample size was around 1000–1200 to achieve meaningful results. Randomization was carried out with computer-generated random numbers in a block (10 participants) randomization pattern. Randomization, as well as treatment concealment was done by the pharmacologist. Out of the 1855 individuals screened for enrolment, 518 individuals were symptomatic. Out of 1337 asymptomatic individuals, 7 individuals were non-COVID suspects, 87 were HCW and, 12 individuals already received COVID-19 vaccine, 4 had suspected ECG abnormality at baseline and 27 individuals did not consent for participation in the study (Fig. 1). Eventually, a total of 1200 participants were enrolled in the trial, out of which 32 individuals could not be followed up completely. Finally, the data of 1168 participants were analyzed, 594 participants in the Control/Placebo group and 574 participants in the PEP/HCQ group (Fig. 1). The data from the individual participants were organized in Microsoft Excel and statistical analysis was executed using SPSS 21.0 version. Evaluation of the parametric data was performed by unpaired t-Test and chi-squire test with yet’s correlation was used to evaluate the binominal/categorical endpoints and Fisher’s exact test was performed for the comparison of the data of proportions. The relative risk (RR) and number needed to treat (NNT) were worked out for the assessment of risk and safety. A p-value of less than 0.05 was considered statistically significant (95% CI).

## Results

### Study population’s baseline characteristics

Out of 1168 participants, majority of the participants were male 865 (74.1%) than female 303 (25.9%). Study population’s mean age was 35.0 (± 10.4) years, with majority of the participants were from 18 to 45 years age group (84.4%), followed by > 45–65 years age group (14.2%), and > 65 years of age group (1.4%). None of the participants had previous history of COVID-19. Out of 1168 participants, 234 (20.0%) individuals were alcohol consumers and 101 (8.6%) individuals were smokers. Diabetes mellitus (N = 43, 3.7%) was the most common co-morbidity, followed by hypertension (N = 41, 3.5%), hypothyroidism (N = 11, 0.9%), coronary artery disease (N = 3, 0.2%) and bronchial asthma (N = 1, 0.08%).The study groups were comparable for the age, age-groups, gender, risk factors and co-morbidity (Table 1).

**Table 1:** Baseline demographic and clinical parameters of the study population.

Parameters	Control group (N = 594)	PEP/HCQ group (N = 574)	P value
Mean age (years ± SD)	35.1 ± 10.4	35.0 ± 10.4	0.845
18–45 years (N = 986)	500 (84.2%)	486 (84.7%)	0.816
> 45–65 years (N = 166)	85 (14.3%)	81 (14.1%)	0.922
> 65 years (N = 16)	9(1.5%)	7 (1.2%)	0.663
Male (N = 865)	428 (72.1%)	437 (76.1%)	0.112
Female (N = 303)	166 (27.9%)	137 (23.9%)	0.112
Smoker (N = 101)	51 (8.6%)	50 (8.7%)	0.939
Alcohol consumer (N = 234)	113 (19.0%)	121 (21.1%)	0.380
Diabetes (N = 43)	23 (3.9%)	20 (3.5%)	0.725
Hypertension (N = 41)	25 (4.2%)	16 (2.8%)	0.187
Coronary artery disease (N = 3)	3 (0.5%)	0(0.0%)	0.088
Hypothyroidism (N = 11)	7 (1.2%)	4 (0.7%)	0.394
Bronchial asthma (N = 1)	0 (0.0%)	1 (0.2%)	0.308

### Primary outcome

A total of 51 out of 1168 participants (4.4%) were diagnosed to have new onset COVID-19 during 4 weeks of follow-up. The incidence of COVID-19 was similar (p = 0.761) in the PEP group [N = 24 out of 574, (4.2%)] which received HCQ prophylaxis and the control group [N = 27 out of 594, (4.5%)] which received Placebo instead of HCQ (Table 2). Total absolute risk reduction for the incidence of new onset COVID-19 in participants of PEP group was -0.3% points as compared to the placebo group and number needed to treat (NNT) was 333.3 with a relative risk of 0.91 (95% confidence interval, 0.52 to 1.60). The NNT was very high (333.3), which indicated that to prevent the incident of one COVID-19 case, at least 333 high risk individuals were required to be treated with HCQ prophylaxis.

**Table 2:** Incidence of COVID-19 after post exposure prophylaxis with HCQ and placebo.

Outcomes	Placebo/Control group (N = 594)	PEP/HCQ group (N = 574)	P value
COVID-19 * (N = 51)	27 (4.5%)	24 (4.2%)	0.761
Definite COVID-19 ^α^ (N = 37)	21 (3.5%)	16 (2.8%)	0.466
Probable COVID-19 ^β^ (N = 14)	6 (1.0%)	8 (1.4%)	0.547
New onset symptoms (N = 41)	24 (4.0%)	17 (3.0%)	0.317
Moderate to severe COVID-19	0	0	-

*The participant with either definite or probable COVID-19 was defined as COVID-19 case.

Total absolute risk reduction was -0.3% points and number needed to treat (NNT) was 333.3 with relative risk of 0.91 (95% confidence interval, 0.52 to 1.60).

^α^The participant with RTPCR positive for SARS-CoV-2 and with or without symptoms was defined as definite COVID-19 case.

Total absolute risk reduction was -0.7% points and the NNT was 143 with relative risk of 0.78 (95% confidence interval, 0.40 to 1.51).

^β^The participant with new onset COVID-19 related symptoms but RTPCR negative for SARS-CoV-2 or could not be performed for any reason was defined as probable COVID-19 case.

Total 37 out of 1168 participants (3.2%) were diagnosed to have definite COVID-19 (RTPCR-positive with or without symptoms) during the 4 weeks of follow-up. Though the incidence of definite COVID-19 was lower in the PEP group [N = 16 out of 574, (2.8%)] as compared to the control group [N = 21 out of 594, (3.5%)], it was not significantly (p = 0.466) different (Table 2). Total absolute risk reduction for the incidence of new onset definite COVID-19 in participants of PEP group was -0.7% points as compared to the placebo group and the NNT was 143 with a relative risk of 0.78 (95% confidence interval, 0.40 to 1.51). The NNT was again very high (143), which indicated to prevent incident of one definite COVID-19 case, at least 143 high risk individuals were required to be treated with HCQ prophylaxis.

Out of 1168 participants, 14 individuals (1.2%) were diagnosed to have probable COVID-19 (symptomatic with RTPCR negative or could not be performed for any reason) during the 4 weeks of follow-up. The incidence of probable COVID-19 was also similar (p = 0.547) both in the PEP group [N = 8 out of 574, (1.4%)] and the control group [N = 6 out of 594, (1.0%)] (Table 2).

The incidence of COVID-19, definite COVID-19 and probable COVID-19 was also similar in different age groups of the study population (Table 3).

**Table 3:** Incidence of COVID-19 after post exposure prophylaxis with HCQ and placebo in different age groups.

Age group	Outcomes	Placebo/control group (N = 594)	PEP/HCQ group (N = 574)	P value
18–45 years (N = 986)	COVID-19* (N = 48)	26 out of 500 (5.2%)	22 out of 486 (4.5%)	0.623
	Definite COVID-19 ^α^ (N = 36)	20 out of 500 (4.0%)	16 out of 486 (3.3%)	0.554
	Probable COVID-19^β^ (N = 12)	6 out of 500 (1.2%)	6 out of 486 (1.2%)	0.961
> 45–65 years (N = 166)	COVID-19* (N = 3)	1 out of 85 (1.2%)	2 out of 81(2.5%)	0.532
	Definite COVID-19 ^α^ (N = 1)	1 out of 85 (1.2%)	0 out of 81 (0.0%)	0.328
	Probable COVID-19^β^ (N = 2)	0 out of 85 (0.0%)	2 out of 81 (2.5%)	0.145
> 65 years (N = 16)	COVID-19* (N = 0)	0 out of 9 (0.0%)	0 out of 7 (0.0%)	-
	Definite COVID-19^α^ (N = 0)	0 out of 9 (0.0%)	0 out of 7 (0.0%)	-
	Probable COVID-19^β^ (N = 0)	0 out of 9 (0.0%)	0 out of 7 (0.0%)	-

*The participant with either definite or probable COVID-19 was defined as COVID-19 case.

^α^The participant with RTPCR positive for SARS-CoV-2 and with or without symptoms was defined as definite COVID-19 case.

^β^The participant with new onset COVID-19 related symptoms but RTPCR negative for SARS-CoV-2 or could not be performed for any reason was defined as probable COVID-19 case.

RT-PCR of nasopharyngeal swabs for SARS-CoV-2 couldn’t be not be performed in 12 (1.0%) participants for diverse reasons which included the stringent lockdown, lack of interest or fear for testing and social stigma. But all of them completed the therapy as per the study protocol and could be followed for 4 weeks to record the outcome. If we consider only the participants (N = 1156) in whom RT-PCR could be performed, even then the incidence of new onset definite COVID-19 was similar (P = 0.460) in the PEP group (N = 16 out of 569, 2.8%) and the control group (N = 21 out of 587; 3.6%) and overall incidence of COVID-19 was also similar (P = 0.968) in PEP (N = 23 out of 569, 4.0%) and the control group (N = 24 out of 587; 4.1%) (eTable 1).

### Symptomatic COVID-19

Out of 51 COVID-19 cases majority of them were symptomatic 41 (80.4%) and only 10 (19.6%) participants were asymptomatic. The incidence of symptoms were comparable (p = 0.317) between the control group [N = 24 out of 594, 4.0%] and PEP [17 out of 574, 3.0%] groups (Table 2). None of the COVID-19 cases progressed to moderate to severe disease, requiring oxygen replacement or life support. Most common symptom was fever (23 out of 51 participants; 45.1%) followed by cough & sore throat in 15 (29.4%) participants, followed by myalgia in 5 (9.8%), and head ache in 3 (5.9%) individuals. Diarrhea, joint pain, body ache and sneezing were complained by 1 (2.0%) participant each and 12 individuals had 2 or more symptoms. The COVID-19-related symptoms were similar in the PEP and control group (eTable 2).

### Secondary outcome

Overall compliance to the therapy was very good. The compliance to therapy was inadequate in 4 (0.3%) participants (1 in control group and 3 in PEP group) who stopped taking medication after day one either due to side effect or the anxiety related to the possible side effect. The drug was well tolerated and no serious adverse events were observed during the study period. Overall, 7 (0.6%) participants reported ADR, which was similar (P = 0.739) in the control (4, 0.7%) and PEP (3, 0.5%) groups. Most common ADR was gastritis-related symptoms in the form of epigastric abdominal discomfort with burning sensation, nausea and vomiting reported after loading dose by 4 participants (2 each from control and PEP group) which resolved with antacid without recurrence thereafter. Itching with skin rash over the foot was noticed in 2 participants (1 each from either group), which resolved spontaneously within few days after discontinuation of the study drug. Episode of palpitation was reported by one participant from PEP group during the follow-up. It was a single episode, self-limiting and lasted for less than a minute, for which no specific treatment was required, and there was no recurrence of episodes of palpitation thereafter.

## Discussion

In this randomized, double-blind placebo-controlled clinical trial, 1168 asymptomatic individuals of high risk household direct contact with the laboratory-proven COVID-19 cases received PEP with either HCQ or Placebo and followed up for 4 weeks for the detection of new onset COVID-19. We found that PEP with HCQ was not associated with significant reduction in incidence of new onset COVID-19 as compared to the Placebo. Vaccines against COVID-19 came with great hope for the prevention of COVID-19 as well as reduction of the severity of illness and subsequent decrease in mortality. Even after more than a year of vaccine roll-out, many countries are going through multiple waves of the pandemic adding further assault to already traumatised human lives. Despite covering more than 66% of the population with full 2-dose vaccination, countries like the USA, UK and India have no respite from the surge of new COVID-19 cases^1^. On the other hand, India, despite administering more than 2048 million doses of vaccine, could be able to cover only about 67% of its population with full vaccination, as of 1st August 2022^1^. It was because of huge number of population, and still around 20 thousands new cases being reported daily in India^1^. As of 28th July 2022, the USA was able to cover more than 32% of the population with booster doses of vaccination, but still reporting more than 100 thousand new cases daily^1^.This was more because of rising numbers of mutated variants of the SARS-CoV-2, rendering neutralizing antibody less effective against it, and decreasing effective vaccine efficacy at the ground level^26,27^. As the majority of COVID-19 patients are asymptomatic, hardly requiring hospitalization, prevention of COVID-19 in high-risk individuals is the upmost priority to restrain this devastating pandemic. An anti-viral drug effective against SARS-CoV-2 is the need of the hour along with mass vaccination to combat this pandemic more efficiently to put an end to the ongoing human misery. Mass production as well as distribution of drugs to the poorest sections of society is much easier than complex vaccine manufacturing leading to better coverage against COVID-19. Oral antiviral drugs, Paxlovid, Molnupiravir received emergency use authorization from the US FDA for mild to moderate COVID-19, as it was found to reduce the risk of hospitalization or mortality^8,9^. But for the prevention of COVID-19, PEP with Paxlovid was found to be ineffective and the role of PEP with Molnupiravir is still inconclusive^8^.

HCQ generated sense of belief for the treatment and prevention of COVID-19 due to its in vitro virucidal action against SARS-CoV-2^28^. Hydroxychloroquine (HCQ), a less toxic derivative of chloroquine (CQ) is part of the 4-aminoquinoline family compounds^29^. HCQ  was initially used as an anti-malarial agent and presently is best recognized as an immunomodulatory and anti-inflammatory agent for the treatment of autoimmune diseases likes rheumatoid arthritis and systemic lupus erythematosus. The proposed primary mechanism for anti-viral action of HCQ is alkalinization of endosomes and lysosomes and subsequent rise in pH leading to inhibition of viral nucleic acid replication, protein glycosylation, viral assembly and transportation, and release of new viral particles^28,29^. Additionally, HCQ can also block viral uptake by inhibiting the glycosylation of the ACE-2 receptor at the plasma membrane to which viral spike protein binds for cellular entry^29^. Further, the immunomodulatory action can also limit the hyperimmune and inflammatory response of COVID-19^30,31^. The Omicron has improved ability to enter cells via endosome and HCQ has the potential against Omicron variant of SARS-CoV-2 through alkalinization of endosomes, preventing membrane fusion. But this hypothesis is yet to be proven by clinical trials.

Presently evidence emerged against the therapeutic benefit of HCQ in hospitalised COVID-19 patients in term of recovery and prevention of mortality^21,22^. But the role of HCQ as PEP for the prevention of COVID-19 remained uncertain when the study was conducted. ICMR data from multiple national institutions, as well as our institutional data were supportive for PEP with HCQ for the prevention of COVID-19^17,18^. There was also lack of RCT, done on Indian population regarding PEP with HCQ. Majority of the studies which produced evidence in favour of HCQ for the prevention and treatment of COVID-19 were either observational or retrospective in nature^13,18,24,25^. So far, Boulware et al. was the largest RCT which concluded that PEP with HCQ was not beneficial for the prevention of COVID-19^21^. However, concerns were raised regarding study design, methodology, and case definition. In this study, the time interval for intervention (exposure & recruitment to 1st dose) was significantly prolonged (> 72 h) in majority of the participants. Delayed intervention may render the drug less efficacious for the prevention of COVID-19 and rather turnout to be more about treatment or prevention of progression of COVID-19^25^. The study population of the present RCT was larger and all the participants received their 1st dose of HCQ within 24 h of proven exposure during their hospital visit with family members or friends for any medical emergency or COVID-19 related work-up. This enabled us, majority of participants to receive their 1st dose under direct observation therapy (DOT) at the time of enrollment itself.

Boulware et al. defined COVID-19 cases as symptomatic illness supported by a positive molecular assay or COVID-19-related symptoms, but asymptomatic presentation of COVID-19 is also very frequent^3^. This was a probable reason for detecting very few number PCR-confirmed COVID-19 cases (16 of 107 symptomatic cases), missing asymptomatic cases. In the present study RTCPR for SARS-CoV-2 RNA was performed in both symptomatic and asymptomatic individuals and was able to detect asymptomatic COVID-19 cases also. According to our study, 20% (10 out of 51) of COVID-19 cases were asymptomatic.

In Boulware et al. the majority (66.4%) of participants were HCW. There is still scarcity of RCT, exploring PEP with HCQ for the prevention of SARS-CoV-2 infection in high-risk household direct contacts of COVID-19 cases. The present study aimed to evaluate the efficacy of HCQ as PEP for prevention of COVID-19, particularly in asymptomatic household direct contacts of the laboratory-confirmed COVID-19 cases. Though, the HCW related to COVID-19 patient care and the household direct contacts of COVID-19 cases are at risk for SARS-CoV-2 infection, they have a comparatively different risk category. HCWs are expected to participate in COVID-19 patient care after wearing personal protective equipments, usually for a fixed duty hours, and can retain a safe distance from the patients as per feasibility. The household contacts get exposure to COVID-19 unknowingly without any precautions or personal protective equipment, as the patients are either family members or friends or relatives, and they live with COVID-19 patients for days to weeks without retaining a safe distance. Therefore, household direct contact possesses a greater risk for COVID-19 compared to HCW and incidence of COVID-19 after exposure may differ in HCW and household direct contact. So the HCW were excluded from the present study.

In a household RCT, Barnabas et al. concluded that PEP with HCQ was not efficacious for the prevention of COVID-19^32^. However, the sample size was smaller; the study did not exclude HCW and more significantly, the time interval from enrollment to the intervention was more than 48 h due to the remote recruitment of participants. The cumulative dose of HCQ was also higher (3400 mg) leading to a higher incidence of ADR.

Additionally, a Cluster-Randomized Trial concluded that PEP with HCQ did not prevent SARS-CoV-2 infection in asymptomatic direct contact of COVID-19 cases^33^. However, it was not a blinded study and majority of the participants received delayed HCQ intervention > 72 h of proven contact. Further published RCT also emphasized that HCQ prophylaxis was not beneficial for the prevention of COVID-19 but again it was done on HCW with very little sample size due to early termination of the study^34^. Another RCT found ineffectiveness of HCQ for prevention of COVID-19 in HCW who received HCQ of 400 mg weekly or twice-weekly^35^. But, this study also raised questions regarding the dose of HCQ for prophylaxis as it achieved very low therapeutic blood concentrations leading to its failure^35^.

Safety has always been a concern against the use of HCQ as it can cause life-threatening cardiac arrhythmias, QT prolongation, retinopathy causing loss of vision, hypoglycaemia, and haemolysis in G6PD-deficient patients. However, these side effects are not common in conventional practice and HCQ has been found to be safe with prolonged lifelong use in rheumatoid arthritis and systemic lupus erythematosus^36,37^. In the present study, none of the participants had serious ADR. The drug was tolerated well in most of the participants with good compliance and was found to be safe. Most commonly reported ADR was mild gastritis-related symptoms on day one or two after receiving 800 mg of loading dose, which responded well with antacid without any recurrence thereafter. Though one participant reported an episode of palpitation, it resolved spontaneously, lasting for less than a minute without any recurrence. The incidence of ADR was higher in the studies by Boulware et al. and Rajasingham et al., probably due to higher cumulative dose (3800 mg and 5600 mg to 10,400 mg respectively) of HCQ compared to the present study (2000 mg)^21,35^.

HCQ was also tried unsuccessfully against the previous corona virus pandemic (MARS) and perhaps COVID-19 is not the last corona virus pandemic either. Despite the unfavorable results of HCQ for the treatment and prevention of COVID-19, the practice of irrational use of HCQ for the prevention of COVID-19 still continues in many countries^31^. The advisory and indiscriminate use of HCQ for COVID-19 had been predominantly under the influence of fear for SARS-CoV-2 infection and social media forces rather than evidence based on clinical research outcomes. Perhaps, this study probably put an end to the era of controversy with HCQ for the prevention of COVID-19. With multiple clinical trials showing evidence against HCQ, the ICMR withdrew HCQ for the management or prevention of COVID-19 in August 2021^38^.

HCQ remains a myth only as it couldn’t produce sufficient in vivo evidence in favor of its benefit against COVID-19. But, at the same time it opens the gate for further research in search of newer and efficacious anti-viral drugs or repurposing of the established drugs against SARS-CoV-2 making us more efficient in defeating this ongoing pandemic. Till then, vaccination, increasing social awareness about the disease and better adherence to the use of face mask, maintaining adequate social distancing and hand hygiene are the best way for the prevention of COVID-19, breaking the chain of transmission of COVID-19 in community^39^.

Limitation of the study was, being a single center clinical trial raised doubt regarding the representation of large diverse Indian population. However, being a tertiary care center, the PGIMER caters to multiple states which include Punjab, Haryana, Himachal Pradesh, Uttarakhand, Uttar Pradesh, Jammu & Kashmir, Rajasthan and Bihar, representing large and diverse parts of India.

The event number (primary end point) was lower than expected. The sample size may have been inadequate to exclude small but clinically meaningful decreases in the incidence of SARS-CoV-2 infection in high risk individuals.

Follow-up ECG could not be done to detect new onset QT prolongation. Only one patient had a self-limiting episode of palpitation without recurrence, and none of the participants reported any symptoms related to any acute cardiac events.

Majority of the participants represented a younger (18–45 years) age group (84.4%) with less representation for older age groups who may have contrasting risk factors for COVID-19 than younger population, and only 3 participants from > 45 years age group had COVID-19 (Table 3). But the incidence of COVID-19 was comparable in different age groups in the present study (Table 3).

In conclusion, PEP with HCQ is not advantageous for the prevention of COVID-19 in asymptomatic household direct contact of the laboratory-confirmed COVID-19 cases. Though HCQ is a safer drug, the practice of irrational and indiscriminate use of HCQ for COVID-19 should be restrained with better pharmacovigilance till further supportive evidence emerges. Till then, mass vaccination, increasing social awareness and use of face masks, social distancing and personal hygiene are the best ways for the prevention of COVID-19.

## Supplementary Information

Supplementary Information.

## Data Availability

All reasonable data requests should be submitted to the corresponding author (DPD) for consideration.
