# Supplementary material for: The ‘myth of Hydroxychloroquine (HCQ) as post-exposure prophylaxis (PEP) for the prevention of COVID-19’ is far from reality
Source: Sci Rep. 2023 Jan 7;13:378. doi: 10.1038/s41598-022-26053-w (PMC9825075; doi:10.1038/s41598-022-26053-w)
Supplement: Supplementary file 1 — Supplementary Information. [file 41598_2022_26053_MOESM1_ESM.pdf]

The 'myth of Hydroxychloroquine (HCQ) as post-exposure prophylaxis (PEP) for the prevention of COVID-19' is far from reality

List of authors:

1. **Dr. Deba Prasad Dhibar**
2. **Dr. Navneet Arora:**
3. **Dr. Deepak Chaudhary**
4. **Dr. Ajay Prakash**
5. **Dr. Bikash Medhi**
6. **Dr. Neeraj Singla**
7. **Dr. Ritin Mohindra**
8. **Dr. Vikas Suri**
9. **Dr. Ashish Bhalla**
10. **Dr. Navneet Sharma**
11. **Dr. Mini P Singh**
12. **Dr. Lakshmi PVM**
13. **Dr. Kapil Goyal**
14. **Dr. Arnab Ghosh**

Legend:

eTable 1: Incidence of COVID-19 after post exposure prophylaxis with HCQ and placebo in participants in whom RTPCR could be performed

eTable 2: COVID-19 related symptoms of the study population

eTable 1: Incidence of COVID-19 after post exposure prophylaxis with HCQ and placebo in participants in whom RTPCR could be performed

Outcomes (N=1156)	Placebo/Control group (N=587)	PEP/HCQ group (N=569)	P value
COVID-19 * (N=47)	24 (4.1%)	23 (4.0%)	0.968
Definite COVID-19 [α] (N=37)	21 (3.6%)	16 (2.8%)	0.460
Probable COVID-19 (N=10)	3 (0.5%)	7 (1.2%)	0.187

* Total absolute risk reduction was -0.1% point with number needed to treat (NNT) was 1000 and relative risk was 0.98 (95% confidence interval, 0.55 to 1.77).

[α] Total absolute risk reduction was -0.8% point with number needed to treat (NNT) was 125 and relative risk was 0.78 (95% confidence interval, 0.40 to 1.51).

eTable 2 : COVID-19 related symptoms of the study population

COVID-19 symptoms	Control group (N=594)	PEP/HCQ group (N=574)	P value
Fever (N=23)	13 (2.2%)	10 (1.7%)	0.583
Sore throat (N=15)	8(1.3%)	7(1.2%)	0.847
Cough (N=15)	8(1.3%)	7(1.2%)	0.847
Myalgia (N=5)	2(0.3%)	3(0.5%)	0.627
Head ache (N=3)	2 (0.3%)	1 (0.2%)	0.583
Diarrhoea (N=1)	0 (0.0%)	1 (0.2%)	.309
Joint pain (N=1)	1 (0.2%)	0 (0.0%)	0.325
Sneezing (N=1)	1 (0.2%)	0 (0.0%)	0.325
Body ache (N=1)	0 (0.0%)	1 (0.2%)	0.309

Study protocol

1. Title: **Efficacy of Hydroxychloroquine (HCQ) as post exposure prophylaxis (PEP) for prevention of COVID-19 in asymptomatic individual at risk for SARS-CoV-2 infection-A randomized control clinical trial**
2. Broad area: **Clinical trial**
3. Duration: **December 2020 onward to March 2021 (ongoing pandemic)**
4. Total estimated budget: **NA (not required at present)**
5. **Principle Investigator**: **Dr. Deba Prasad Dhibar**

Assistant Professor, Department of **Internal Medicine**, PGIMER, Chandigarh

Email: drdeba_prasad@yahoo.co.in, Mobile number: 9530881462

6. **Detail of Co-investigator:**

i) **Dr. Neeraj Singla,** Assistant Professor, **Internal Medicine**, PGIMER, Email: neearjsingladr@yahoo.com, Mobile: 9646121641

ii) **Dr. Ritin Mohindra,** Assistant Professor, **Internal Medicine**, PGIMER, Email: ritin.mohindra@gmail.com, Mobile: 9818700713

iii) **Dr. Vikas Suri,** Additional Professor, **Internal Medicine (Infectious disease)**, PGIMER, Email: surivikas9479@gmail.com , Mobile: 7087009683

iv) **Dr. Ashish Bhalla,** Professor, **Internal Medicine (Infectious disease)**, PGIMER, Email: bhalla.chd@gmail.com, Mobile: 9417023973

v) **Dr. Navneet Sharma,** Professor, **Internal Medicine (Infectious disease)**, PGIMER, Email: navneet207@gmail.com , Mobile: 9417023973

vi) **Dr. Mini P Singh,** Professor, **Virology**, PGIMER, Chandigarh Email: singh.minip@pgimer.edu.in, Mobile:7087008173

vii) **Dr. Lakshmi PVM**, Professor, Community Medicine & School of Public Health PGIMER, Email: pvm.lakshmi@yahoo.com, Mobile:9872628236

viii) **Dr. Bikash Medhi,** Professor, **Pharmacology**, PGIMER Email: drbikashus@yahoo.com, Mobile: 9815409652

7. **Project summary:**

Novel corona virus (SARS-CoV-2) epidemic which started from Wuhan in China is now a well established pandemic affecting more than 57 million people worldwide with more than 1.3 million of mortality. COVID-19 affected more than 9 million of people with more than 130 thousand death in India. If adequate preventive and therapeutic measures are not taken, India has very high risk of affecting million of more people with high mortality because of the large population along with very high population density. At present there are no definitive therapeutic drugs or vaccine available for the treatment and prevention of SARS-CoV-2 infection. Symptomatic and supportive care are being given to COVID-19 cases along with isolation and quarantine measure are being taken for the suspected individual at risk for COVID-19 to limit the spread of the SARS-CoV-2 infection . Among the all the drugs being used for the treatment of COVID-19, hydroxychloroquine (HCQ), has given some rays of hope to battle against this deadly pandemic. HCQ has some anti viral effect against SARS-CoV in vitro. HCQ is quite safe and being used in rheumatology patients for lifelong without much side effect, so it allow for higher dose without any significant side effects and drug-drug interaction. Recently published clinical trial suggested HCQ can be used for the therapeutic purpose of the SARS-CoV-2 infection and many governments have endorsed that due to lack of any other better alternative drugs. Indian council of medical research (ICMR) has advised for HCQ prophylaxis for the people who are at risk for developing SARS-CoV-2 infection, all asymptomatic health care workers involved in taking care of suspected or confirmed COVID-19 cases and all asymptomatic household contacts of laboratory confirmed COVID-19 cases. This encouraged us to conduct an open level clinical trial on HCQ as post exposure prophylaxis (PEP) for the prevention of COVID-19 in asymptomatic high risk house hold contact of the laboratory confirmed COVID-19 cases. The result was very promising showing absolute risk reduction of around 9% in participant who received PEP with HCQ as compared to the control group and there was no serious adverse event. But there is still conflicting scientific data to prove or disprove the efficacy of HCQ for the treatment and prophylaxis for SARS-CoV-2 infection. Being a tertiary care centre we are catering many states which include Punjab, hariyana, himachal Pradesh, Uttara khand, Uttar Pradesh. This put our institute to handle highest burden of suspected cases of SARS-CoV-2 in northern India. So we have subsequently planned this double blind clinical trial to evaluate the efficacy of HCQ as PEP for the prevention of COVID-19 in asymptomatic individuals who are at risk for SARS-CoV-2 infection. As a research institute of national as well as international interest, it is a great opportunity for us to produce such a robust data which can be utilized in reforming national and international guidelines for the battle against of COVID-19 pandemic. The asymptomatic individual with direct contact with laboratory confirmed COVID-19 cases will be randomized into one PEP group and one control/placebo group as per inclusion and exclusion criteria. Individual who will not give consent for HCQ prophylaxis and those

with contraindication for HCQ therapy like, hypersensitivity to HCQ or 4-aminoquinolone derivatives, patients with known retionopathy, cardiac arrhythmia, G6PD deficiency, psoriasis and pregnancy will be excluded from the study. All symptomatic individual and all health care workers related to suspected or proven COVID-19 and who received CoVID-19 vaccine will be excluded from the study. The PEP group will receive tablet HCQ 400 mg q 12 hourly on day one followed by 400 mg once weekly for 3 weeks (total cumulative dose of 2000 mg). The control group will receive placebo instead of HCQ. Both the groups will receive standard care of therapy in the form of home quarantine for 2 weeks along with social distancing and personal hygiene. They will be followed up for 4 weeks telephonically or physically as and when required and will be enquired regarding development of any COVID-19 symptoms like fever, cough, sore throat, shortness of breath, diarrhoea, myalgia. During follow up nasopharyngeal swab of the participants will be taken for processing reverse transcription polymerase chain reaction (RTPCR) for the detection of SARS-CoV-2 RNA to confirm the CoVID-19. Samples for RTPCR will be taken when any asymptomatic participants becomes symptomatic and by the 5-14 days of contact in asymptomatic participants through in-hospital visit at the institute's COVID-19 screening clinic at Emergency medical out patients department (EMOPD) or communicable disease ward isolation. The participants with new onset COVID-19 will be managed as per institute's COVID-19 management protocol and the participants needing admission will be given best possible medical care in the institute's SARI/Nehru extension COVID ward under isolation. Only the participant with RTPCR positive for SARS-CoV-2 and with or without symptoms will be defined as definite COVID-19 case. The participant with new onset symptoms, but RTPCR negative for SARS-CoV-2 or cannot be performed for any reason will be defined as probable COVID-19 case. Asymptomatic participants with negative RTPCR will be defined as non-COVID case. The incidence of COVID-19 will be compared between the PEP and control groups. Department of internal medicine (Emergency) will be taking care of screening, enrolment, patients management, follow up and data analysis. Department of pharmacology will take care of randomization & blinding and adverse events monitoring. Department of Virology will take care of molecular diagnosis of COVID-19 by RTPCR as and when required. Department of Community medicine & school of public health will take care of contact tracing and information to the local IDSP.

Certification: "I certify that I will follow the prescribed/established Ethical Norms of regulatory bodies, ICMR and as defined in the SOP of IEC, PGIMER. Further, the project which is being submitted has not been plagiarized from any source."

Signature of the PI:

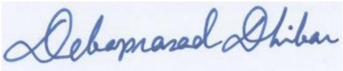

Name: Deba Prasad Dhibar

Part-II

1. **Research hypothesis:**

There are no definitive proven therapeutic drugs for SARS-CoV-2 infection management. Among the various drugs used for the treatment of SARS-CoV-2, HCQ has given some rays of hope to battle against this deadly pandemic due to its anti viral effect against SARS-CoV in vitro. Indian council of medical research (ICMR) has advised for HCQ prophylaxis for the people who are at risk for developing SARS-CoV-2 infection, all asymptomatic health care workers involved in taking care of suspected or confirmed COVID-19 cases and all asymptomatic household contacts of laboratory confirmed COVID-19 cases. This encouraged us to conduct an open level clinical trial on HCQ as post exposure prophylaxis (PEP) for the prevention of COVID-19 in asymptomatic high risk house hold contact of the laboratory confirmed COVID-19 cases. The result was very promising showing absolute risk reduction of around 9% in participant who received PEP with HCQ as compared to the control group and there was no serious adverse event. But there is still conflicting scientific data to prove or disprove the efficacy of HCQ for the treatment and prophylaxis for SARS-CoV-2 infection. This study will evaluate the eefficacy of HCQ as PEP to prevent SARS-CoV-2 infection in asymptomatic at risk individual.

2. **Background and rational of the study:**

Novel corona virus (SARS-CoV-2) epidemic which stared from Wuhan in China is now a well established pandemic affecting more than 57 million people worldwide with more than 1.3 million of mortality [1]. After Italy, Spain, Germany, United Kingdom and Brazil have taken over from China in term of the burden of mortality, India is the next epicentre of this pandemic just behind USA. COVID-19 affected more than 9 million of people with more than 130 thousand of death in India [2]. The clinical presentation of the COVID-19 varies from asymptomatic cases and mild symptoms of fever, cough, sore throat, headache, myalgia, nasal congestion, diarrhea to severe pneumonia, acute respiratory distress syndrome (ARDS) requiring mechanical ventilation and even multi-organ dysfunction syndrome (MODS), sepsis leading to death [3]. If adequate preventive and therapeutic measures are not taken, India has very high risk of affecting million of people with high mortality because of the large population, very

high population density and ongoing relaxation of the previously imposed lockdown. At present there are no definitive therapeutic drugs or vaccine available for the treatment and prevention of SARS-CoV-2 infection. Symptomatic and supportive care are being given to COVID-19 cases along with isolation and quarantine measure are being taken for the suspected individual at risk for COVID-19 to limit the spread of the SARS-CoV-2 infection [4].

Presently many scientist and doctors are recommending many existing available drugs (Ribavirin, lopinavir, Remdesivir, chloroquine, hydroxychloroquine) for SARS-CoV-2 infection for therapeutic and as well as prophylactic purpose [5,6]. Among which hydroxychloroquine sulphate (HCQ), a chloroquine analogue has given some rays of hope to battle against this deadly pandemic [7]. In vitro studies showed that HCQ had potent anti viral effect against SARS-CoV through mechanism targeted at the host cell [8-10]. HCQ is quite safe drugs as it is being used in rheumatology patients for lifelong therapy without much side effect, so it allow for higher dose without any significant side effects and drug-drug interaction [11]. Recently published clinical trial suggested HCQ can be used for the therapeutic purpose of the SARS-CoV-2 infection [7]. ICMR has advised for HCQ prophylaxis for the people who are at risk for developing SARS-CoV-2 infection, all asymptomatic health care workers involved in taking care of suspected or confirmed COVID-19 cases and all asymptomatic household contacts of labarotory confirmed COVID-19 cases [12]. This encouraged us to conduct an open level clinical trial on HCQ as PEP for the prevention of COVID-19 in asymptomatic high risk house hold contact of the laboratory confirmed COVID-19 cases. The result was very promising showing absolute risk reduction of around 9% for prevention of COVID-19 in participant who received PEP with HCQ as compared to the control group which did not receive HCQ and there was no serious adverse event [13]. But there is still conflicting scientific data to prove or disprove the efficacy of HCQ for the treatment and post exposure chemo-prophylaxis for SARS-CoV-2 infection management. Recently published study proposed that HCQ was not useful as post exposure prophylaxis (PEP) for prevention of COVID-19 in at risk individual [14]. But in this study majority of the participants were health care workers (HCW). Recently newer studies have come in support of HCQ for the potential preventive measure against COVID-19 [13, 15].

The household direct contacts of the COVID-19 patients are at the highest risk for SARS-CoV-2 infection. There is still lack of clinical trial regarding PEP with HCQ for the prevention of COVID-19 in non-HCW individuals who are at risk of SARS-CoV-2 infection. Being a tertiary care centre we are catering many states which includes Punjab, Haryana, Himachal Pradesh, Uttara Khand, Uttar Pradesh. This put our institute to handle highest burden of suspected cases of SARS-CoV-2 in northern India. So we have planned this randomised control clinical trial to evaluate the efficacy of HCQ as PEP for the prevention of COVID-19 in asymptomatic population who are at risk for SARS-CoV-2 infection. As a research institute of national as well as international interest, it is a great opportunity for us to produce such a robust data which can be utilized in reforming national and international guidelines for the battle against of COVID-19 pandemic.

3. **Preliminary work done:**

Recently we conducted an open level clinical trial on HCQ as PEP for the prevention of COVID-19 in asymptomatic high risk house hold contact of the laboratory confirmed COVID-19 cases. The result was very promising showing absolute risk reduction of around 9% for prevention of COVID-19 in participant who received PEP with HCQ as compared to the control group which did not receive HCQ and there was no serious adverse event [13]. This encourages us to conduct a double blind clinical trial on HCQ as PEP for the prevention of COVID-19 in at-risk individuals. As this is an ongoing serious pandemic without any definitive therapeutic cure and preventive vaccines are still awaited this study purpose to generate adequate data at earliest, which can be utilized in reforming national and international guidelines for the battle against of COVID-19 pandemic.

4. **Relevance clinical importance and expected outcome:**

The study will enlighten us regarding efficacy of HCQ as PEP for the prevention of COVID-19 in asymptomatic individuals who are at risk for SARS-CoV-2 infection. As a research institute of national as well as international interest, it is a great opportunity for us to produce such a robust data which can be utilized in reforming national and international guidelines for the battle against of SARS-CoV-2. As per our recently conducted open level clinical trial there was absolute risk reduction of around 9% for

prevention of COVID-19 in participant who received PEP with HCQ as compared to the control group which did not receive HCQ and there was no serious adverse event [13].

5. **Objectives:**

Primary objectives: To evaluate the efficacy of Hydroxychloroquine (HCQ) as post exposure prophylaxis (PEP) for the prevention of COVID-19 in asymptomatic individuals who are at risk for SARS-CoV-2 infection.

Secondary objectives:

1. New onset symptoms of COVID-19
2. Compliance to the therapy
3. Difficulty faced during quarantine
4. Adverse drug reaction monitoring

6. **Details of method:**

Aims of the study: To evaluate the efficacy of Hydroxychloroquine (HCQ) as post exposure prophylaxis (PEP) for the prevention of COVID-19 in asymptomatic individuals who are at risk for SARS-CoV-2 infection.

Site of Study: The study will be conducted in the special COVID-19 screening clinic at Emergency medical outpatient department (EMOPD) and Communicable disease ward of the Post Graduate Institute of Medical Education and Research (PGIMER), Chandigarh, India. The study will be done under the collaboration of the department of Internal Medicine, Virology, Pharmacology and Community medicine & School of public health of the institute, PGIMER, Chandigarh.

Study Design: It is randomized control double blind clinical trial. The study will be done in two groups. After screening of the asymptomatic individual with direct contact with laboratory confirmed COVID-19 cases will be randomized into one PEP group and one control/placebo group as per inclusion and exclusion criteria. Post exposure prophylaxis (PPE) group will receive tablet HCQ 400 mg q 12 hourly on day one followed by 400 mg once weekly for 3 weeks (total cumulative dose of 2000 mg). The control group will receive placebo instead of HCQ. Both the groups will receive standard care of therapy in the

form of home quarantine for 2 weeks along with social distancing and personal hygiene. The participants will be followed up for 4 weeks telephonically or physically as and when required.

Study duration: Study will be carried out during December 2020 onward to March 2021 (ongoing pandemic).

Screening and Enrolment of Patients: At-risk individuals presenting at COVID-19 screening clinic and through telephonic consultation will be screened for the enrollment in the study as per pre-defined inclusion and exclusion criteria. The asymptomatic individuals with high risk direct contact are family members, relatives, friends or colleagues who are living with or spend hours/days with the COVID-19 patients without taking any personal protective precautions. After screening of the asymptomatic individual with direct contact with laboratory confirmed COVID-19 cases will be randomized into one PEP group and one control/placebo group after getting informed consent.

Inclusion and exclusion criteria:

Irrespective of gender and age of ≥18 years, all asymptomatic individual with direct contact with laboratory confirmed COVID-19 cases will be included in the study. Individual who will not give consent for HCQ prophylaxis and patients with contraindication for HCQ therapy like, hypersensitivity to HCQ or 4-aminoquinolone derivatives, patients with known retionopathy, cardiac arrhythmia, G6PD deficiency, psoriasis and pregnancy will be excluded from the study. Also the symptomatic individual, all health care workers related to suspected or confirmed COVI-19 and who received COVID-19 vaccine will be excluded from the study.

Method and intervention: In this randomized control clinical trial after screening and enrolment, all the at-risk asymptomatic participants will be assigned into one PEP group and one control group as per inclusion and exclusion criteria after getting informed consent. The PEP group will receive tablet HCQ 400 mg q 12 hourly on day one followed by 400 mg once weekly for 3 weeks (total cumulative dose of 2000 mg). The control group will receive placebo instead of HCQ. Both the groups will be advised standard care of therapy in the form of home quarantine for 2 weeks along with social distancing and personal hygiene. The prophylactic dose of HCQ is decided as per ICMR recommendation for PEP with

HCQ [12]. The potential antiviral and anti-inflammatory properties of HCQ, along with the low cost, very good oral bioavailability, higher concentrations in the lungs relative to the plasma levels and acceptable safety profile supported the formulation this national advisory. After getting informed consent the drugs will be dispensed directly to the participants during the COVID-19 screening clinic vist or through door step delivery by the concerned HCW with help of the police officials. They will be followed up for 4 weeks telephonically or physically when require and will be enquire regarding development of any COVID-19 symptoms like fever, cough, sore throat, shortness of breath, diarrhea, myalgia or any adverse drug event. During follow up nasopharyngeal and or throat swab of the participants will be taken for processing reverse transcription polymerase chain reaction (RTPCR) for the detection of SARS-Cov-2 RNA to confirm the diagnosis of CoVID-19. Samples for RTPCR will be taken when any asymptomatic participants become symptomatic and by the 5-14 days of contact in asymptomatic participants through in-hospital visit at COVID-19 screening clinic at EMOPD and the institute's communicable disease ward isolation. Only the participant with RTPCR positive for SARS-CoV-2 and with or without symptoms will be defined as definite COVID-19 case. The participant with new onset symptoms, but RTPCR negative for SARS-CoV-2 or cannot be performed for any reason will be defined as probable COVID-19 case. Both the definite and probable COVID-19 cases together are defined as COVID-19 case. Asymptomatic participants with negative RTPCR will be defined as non-COVID case. Incidence of COVID-19, definite COVID-19 and probable COVID-19 in previously asymptomatic participants will be compared between the PEP and control groups. Baseline routine investigations (blood, CXR, ECG) at upfront may not be possible as each participant will be the potential source of SARS-CoV-2 and additional contact with HCW will spread the virus to the healthy individual. ECG will be offered to each participant at baseline and at the end of the study. Participants will be interviewed during the follow up for any adverse events and ECG will be repeated as per indication. During follow up if any individuals become symptomatic then all relevant investigations will be done as per indications. The participants, who turned out to be definite COVID-19, were shifted to the Nehru Hospital Extension (NHE), a dedicated COVID-19 centre, of the institute PGIMER, Chandigarh and managed as per institutional COVID-19 protocol. Participant

with Probable COVID-19 were advised to continue with home quarantine. The participants with new onset COVID-19 will be managed as per institute's COVID-19 management protocol and the participants needing admission will be given best possible medical care in the institute's SARI/Nehru extension COVID ward under isolation.

Primary outcome: Incidence of COVID-19, definite COVID-19 & probable COVID-19

Secondary outcome: 1. New onset symptoms of COVID-19, 2. Compliance to the advised therapy, 3. Number of difficulty faced during quarantine, 3. Incidence of adverse drug reaction

Sample size & statistical analysis:

As per recently concluded our institutional pilot study, the incidence of COVID-19 was significantly (P = 0.033) lower in the PEP group (10.6%) that received HCQ prophylaxis compared with the control group (19.5%). The total absolute risk reduction for the incidence of COVID-19 in participants received PEP with HCQ was –8.9% points [13]. Anticipating at least 20% incidence of COVID-19 in control group as compared to 10% incidence rate in PEP group the total sample size will be around 400 (200 each group), when there will be 80% (power) chance of detecting a significant difference between the two groups at a one-sided 0.05 significance level (α-error of 5%). Anticipating 10% dropout we will try to recruit total 440 participants for the study. However as per protocol, after following strict inclusion and exclusion criteria the final sample size for the data analysis may reduce further. Final power of the study many be recalculated after getting actual response/outcome at the end of the clinical trial through post-hoc statistical power analysis. Randomization will be done with the help of compute generated random number in a block randomization pattern in a block of 10 patients (5:5). Randomization and treatment concealment will be done by the pharmacologist. The data will be managed in data base system through Microsoft Excel and statistical analysis will be performed by SPSS 21.0 version. The parametric data will be

analyzed by paired or unpaired “t”-test and the binominal/categorical endpoints will be analyzed with non-parametric chi-squire test with yet’s correlation and the proportions will be compared by Fisher’s exact test. The relative risk (RR) and number needed to treat (NNT) will be determined for the safety and risk assessment. The p-value less than 0.05 will be considered statistical significant (95% CI).

Ethical justification: Being a tertiary care centre we are catering many states which include Punjab, hariyana, himachal Pradesh, Uttarakhand, Uttar Pradesh. This put our institute to handle highest burden of suspected cases of SARS-CoV-2 in northern India. At present there is no definitive therapeutic drugs or vaccine are available for the treatment of SARS-C0V-2. Among all the used drugs HCQ has given some rays of hope to battle against this deadly pandemic. Prolong use of HCQ has been found to be quite safe and we are giving HCQ for 3 weeks only and that too weekly with total cumulative dose of 2000 mg only. Our own institutional study showed that HCQ has the potential for the prevention of COVID-19 in house direct contacts of laboratory confirmed COVID-19 cases [13]. All the patients will receive standard best possible medical therapy. The study is not intended to do any invasive procedure or increase the cost of therapy. Study is intended to prevent emergence of SARS-CoV-2 infection in asymptomatic patients with chemo-prophylaxis. Only those individual with high risk for developing infection will be included after getting informed consent. As a research institute of national as well as international interest, it is a great opportunity for us to produce such a robust data which can be utilized in reforming national and international guidelines for the battle against of SARS-CoV-2 pandemic.

The following ethical considerations will be adhered to during the study:

1. Patients will be enrolled in the study after full informed consent has been taken. The patients will have the right to quit the study at any point in time during the study and no compulsion will be exerted on them.

2. In case the patient is unable to consent due to poor neurological status, informed consent will be taken from a close relative available at that time. Patient/legal representative will be informed about the aim and method of the study, investigations that will be conducted on him/her, possible adverse effects associated

with the treatment modality under study and will have full right to refuse any investigation at any point in time during the study even after he/she has signed the consent and he/she will be excluded from the study.

4. The patient, irrespective of whether enrolled, will receive treatment as per the standard protocol followed in the Emergency Department of PGIMER, Chandigarh. The patient will not be exposed to any additional risk or cost.

5. Confidentiality of data collected from contribution source or individual will be maintained.

6. In the publication of the results of this study, all efforts would be made to preserve the accuracy of both the positive and negative results of this study.

References:

1. WHO Coronavirus Disease (COVID-19) Dashboard. [Available from: https://covid19.who.int/]
2. **COVID-19 Dashboard. [Available from: https://www.mygov.in/covid-19/]**
3. Guan W, Ni Z, Hu Y, Liang W, Ou C, He J, Liu L, Shan H, Lei C, Hui DSC, Du B, Li L et al., for the China Medical Treatment Expert Group for Covid-19. Clinical Characteristics of Coronavirus Disease 2019 in China. N Engl J Med. 2020; 382:1708-20. DOI: 10.1056/NEJMoa2002032.
4. Baden LR, Rubin EJ. Covid-19 -The Search for Effective Therapy. N Engl J Med. 2020; 382:1851-52. DOI: 10.1056/NEJMe2005477.
5. Sarma P, Prajapat M, Avti P, Kaur H, Kumar SK, Medhi B. Therapeutic options for the treatment of 2019-novel coronavirus: An evidence-based approach. Indian Journal of Pharmacology. 2020; 52(1):1-5.
6. Wang M, Cao R, Zhang L, Yang X, Liu J, Xu M, *et al*. Remdesivir and chloroquine effectively inhibit the recently emerged novel coronavirus (2019-nCoV) *in vitro*. Cell Res. 2020; 30: 269-71. doi: 10.1038/s41422-020-0282-0.

7. Gautret P, Lagier J, Parola Pa,b, Hoang VT, Meddeb L, Mailhe M, Doudier B, Courjon Je et al. Hydroxychloroquine and azithromycin as a treatment of COVID-19: results of an open-label non-randomized clinical trial. International Journal of Antimicrobial Agents. 2020:105949. DOI: 10.1016/j.ijantimicag.2020.105949.
8. Biot C, Daher W, Chavain N, Fandeur T, Khalife J, Dive D, et al. Design and synthesis of hydroxyferroquine derivatives with antimalarial and antiviral activities. J Med Chem. 2006; 49:2845-49.
9. Yao X, Ye F, Zhang M, Cui C, Huang B, Niu P, et al. In Vitro Antiviral Activity and Projection of Optimized Dosing Design of Hydroxychloroquine for the Treatment of Severe Acute Respiratory Syndrome Coronavirus 2 (SARS-CoV-2). Clin Infect Dis. 2020; ciaa237. doi:10.1093/cid/ciaa237.
10. Vincent MJ, Bergeron E, Benjannet S, Erickson BR, Rollin PE, Ksiazek TG, Seidah NG, Nichol ST. Chloroquine is a potent inhibitor of SARS coronavirus infection and spread. Virology Journal. 2005, 2:69. doi: 10.1186/1743-422X-2-69.
11. Marmor MF, Kellner U, Lai TY, Melles RB, Mieler WF; American Academy of Ophthalmology. Recommendations on Screening for Chloroquine and Hydroxychloroquine Retinopathy. Ophthalmology. 2016; 123(6):1386-94.
12. ICMR. Advisory on the use of hydroxy-chloroquine as prophylaxis for SARS-COV2 infection 2020.[Availablefrom:https://www.mohfw.gov.in/pdf/AdvisoryontheuseofHydroxychloroquinasprophylaxisforSARSCoV2infection.pdf.
13. Dhibar DP, Arora N, Kakkar A, Singla N, Mohindra R, Suri V, Bhalla A, Sharma N, Singh MP, Prakash A, PVM L, Medhi B. Post-exposure prophylaxis with hydroxychloroquine for the prevention of COVID-19, a myth or a reality? The PEP-CQ Study. International Journal of Antimicrobial Agents. 2020. https://doi.org/10.1016/j.ijantimicag.2020.106224.
14. Boulware DR, Pullen MF, Bangdiwala AS, Pastick KA, Lofgren SM, Okafor EC, Skipper CP, Nascene AA, Nicol MR, Abassi M, Engen NW, Cheng MP, LaBar D, Lother SA, MacKenzie LJ, Drobot G, Marten N, Zarychanski R, Kelly LE, Schwartz IS, McDonald EG, Rajasingham R, Lee

TC, Hullsiek KH. A Randomized Trial of Hydroxychloroquine as Postexposure Prophylaxis for Covid-19. N Engl J Med. 2020; 383:517-25DOI: 10.1056/NEJMoa2016638.

15. Chatterjee P, Anand T, Singh KhJ, Rasaily R, Singh R, Das S , Singh H, Praharaj I , Gangakhedkar RR, Bhargava B, Panda S. Healthcare workers & SARS-CoV-2 infection in India: A case-control investigation in the time of COVID-19. Indian J Med Res.2020;151: pp459-467. DOI: 10.4103/ijmr.IJMR_2234_20.

7. Time line:

Study will be carried out during December 2020 onward to March 2021 (ongoing pandemic). As it is an ongoing serious pandemic without any definitive therapeutic cure we will try to generate adequate data at earliest to achieve our objectives at earliest.

8. Updated CV including publication of the principal investigator:

Copy attached

9. **List of intramural project granted:** Ongoing **/ No financial grant received from PGIMER**

i) Title: Efficacy of Empirical Ciprofloxacin plus Metronidazole and Cefixime plus Metronidazole Therapy for the Treatment of Liver Abscess: A Randomised Controlled Clinical Trial **(INT/IEC/2019/000516)**

10. **List of extramural project granted:** Completed/ Funded by Pharmaaz India Pvt. Ltd.

Title: "A prospective, multi-centric, randomized, double-blinded, parallel, saline controlled phase II safety and efficacy study of PMZ-2010 as resuscitative agent for hypovolemic shock due to excessive blood loss to be used along with standard shock treatment." (Ledger page No:2760)

Study design

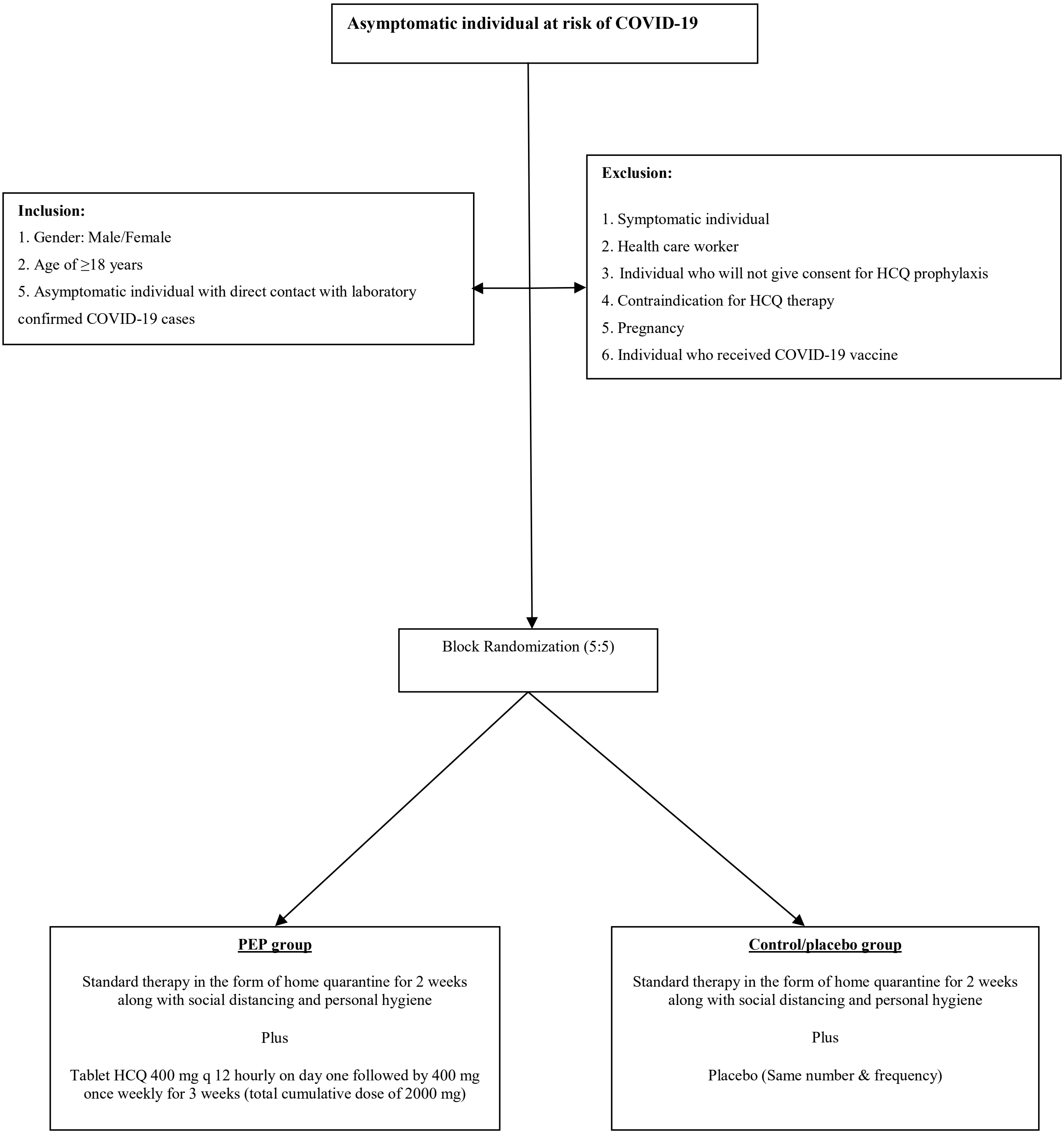

Institutional Ethics Committee

Postgraduate Institute of Medical Education and Research, Chandigarh

Prof. Sanjeev Handa
Chairman

Prof. Nandita Kakkar
Convener

Chairman

Prof. Sanjeev Handa

Members

Dr. S Radhika

Dr. P Kumar

Dr. R Minz

Dr. Rashmi Bagga

Dr. Vishali Gupta

Dr. Devi Dayal

Dr. Neelam Aggarwal

Dr. Ramandep S. Virk

Dr. Vikas Suri

Dr. Amol N Patil

Convener

Prof. Nandita Kakkar

Contact
Office:
+91 172 2755266
Ph:
+91 172 2755141

No: INT/IEC/2021/SPL456
Date:-16/3/21

Dr. Deba Prasad Dhibar,
Dept. of Internal Medicine,
PGIMER, Chandigarh.

Title: **Efficacy of hydroxychloroquine (HCQ) as post exposure prophylaxis (PEP) for prevention of COVID-19 in asymptomatic individual at risk for SARS-CoV-2 infection- a randomized control clinical trial.**

Reference No: NK/6903/Study/101

Dear Dr. Dhibar,

The Institute Ethics Committee (Intramural) at their meeting held on 09.02.2021 has **APPROVED** your above mentioned study protocol after consideration. This meeting was conducted online through Google Meet platform because of COVID-19 pandemic crisis and consequent restrictions.

Note: - The following terms are mandatory, since the study is a randomized controlled trial (If applicable):-

- Kindly get the CTRI registration done
- Please make a Departmental 'data safety monitoring board (DSMB) 'and record the adverse events. The report of this should be sent to the IEC within 24 hours.
- Please send a copy of the DSMB to the IEC soon after getting this letter.

Note: - Dr. Vikas Suri, member of the Ethics Committee (Intramural) is CO-PI in the above mentioned protocol. Therefore, he did not participate when this protocol was being discussed.

It is understood that the study will be conducted strictly as per the submitted protocol. Any deviations from the approved protocol and study documents must be sent to the Ethics Committee for re-approval. Any adverse reaction or condition noted during the study period should be reported to the Ethics Committee immediately.

In all future correspondence with respect to your protocol, please attach a photocopy of this letter.

With kind regards,

Yours sincerely,

Kakkar

(Prof. Nandita Kakkar)
Convener, Instt. Ethics Committee (Intramural),
PGIMER, Chandigarh.

Copy to: Medical Education Cell, PGIMER, Chandigarh.
